# Decoding burn trauma: biomarkers for early diagnosis of burn-induced pathologies

**DOI:** 10.1186/s40364-024-00707-5

**Published:** 2024-12-23

**Authors:** Fadi Khalaf, Daniella Touma, Alexandra Pappas, Lareina Hatim, Stephanie Wojtowicz-Piotrowski, Marc G. Jeschke

**Affiliations:** 1 David Braley Research Institute, Hamilton, ON Canada; 2 Hamilton Health Sciences, Hamilton, ON Canada; 3 Department of Biochemistry, McMaster University, Hamilton, ON Canada; 4 Department of Surgery, McMaster University, Hamilton, ON Canada; 5 David Braley Research Institute, C5-104, 20 Copeland Ave, Hamilton, ON L8L 2X2 Canada

**Keywords:** Burns, Thermal injury, Hypermetabolism, Inflammation, Immunopathy, Sepsis, Biomarkers, Complications, Multiorgan damage

## Abstract

Burn injuries represent a significant global challenge due to their multifaceted nature, characterized by a complex cascade of metabolic and immune dysfunction that can result in severe complications. If not identified and managed promptly, these complications can escalate, often leading to fatal outcomes. This underscores the critical importance of timely and precise diagnosis. Fortunately, biomarkers for burn-induced pathologies and outcomes have emerged as powerful diagnostic and prognostic tools. These biomarkers enable early diagnosis and intervention, facilitate risk assessment, support patient-specific treatment, monitoring of disease progression, and therapeutic efficacy, ultimately contributing to improved patient outcomes. However, while previous studies have provided valuable biomarkers for the detection of burn-induced pathologies, many of these were constrained by the techniques and sample sizes available at the time, which can limit the generalizability of the findings. This review highlights numerous biomarkers studied in the literature to date, underscoring the need to replicate these findings in more diverse and representative populations. It also emphasizes the importance of advancing research efforts to develop more efficient, accurate, and cost-effective approaches for integrating biomarkers into clinical practice.

## Introduction

Burn injuries pose a major global health challenge, affecting nearly 9 million people annually, with 115,000 succumbing to their injuries [1]. In fact, this translates to a person suffering a burn injury every three seconds, and a life being lost to a burn injury every four minutes. Unfortunately, despite advancements in burn wound care, nutrition, fluid resuscitation, and infection control practices in recent decades, morbidity and mortality rates among burn patients remain high [2].

Aside from the visible damage to the dermal layer, burn injuries, especially severe burns, trigger a complex cascade of immune and inflammatory responses, along with significant metabolic alterations [3]. Unlike other forms of trauma, this disruption in homeostasis can persist for years after the injury, potentially leading to complications such as distributive shock, multiple organ failure (MOF), increased risk of infection, sepsis, and ultimately, death [3, 4]. Thus, clinicians face numerous challenges in managing the care of burn patients and must address burn-induced pathologies in a timely and appropriate manner to optimize patient outcomes.

Interestingly, biomarkers for burn-induced pathologies and outcomes have emerged as powerful diagnostic and prognostic tools. These biomarkers play a supportive role in early diagnosis and intervention, aiding in risk assessment, identification of physiological dysfunction, patient-specific treatment planning, and monitoring of disease progression and therapeutic efficacy, thereby contributing to improved patient outcomes. Furthermore, while simple prognostic scoring systems based on factors such as age and burn area are already in use, advancing outcomes further may require addressing poorly understood aspects of the pathophysiological response to burn injury. Incorporating biomarkers into clinical practice may allow for efficient identification of conditions linked to poor outcomes and uncovering mechanisms that could inform targeted therapeutic interventions, ultimately improving overall burn prognosis.

In recent years, there has been a considerable amount of research on trauma biomarkers. Indeed, substantial efforts have been made by researchers to understand how these biomarkers can indicate trauma severity, facilitate early diagnosis, guide treatments and therapies, and predict patient outcomes. In this review, we explore the biomarkers associated with burn-induced pathologies, examining their release patterns, mechanisms of action within the body, and the range of outcomes that result from their release. Additionally, we address gaps in the current literature, highlighting the limitations of these biomarkers as diagnostic tools and discussing areas where further research is needed to enhance their clinical utility and improve patient outcomes.

This review is a narrative literature review, drawing on peer-reviewed articles that focus on the adult burn patient population. In cases where such studies were lacking, we supplemented the review with findings from preclinical studies and other populations to support the evaluation of biomarker efficacy. We utilized various query combinations with targeted keywords such as “biomarker,” “burn,” “burn injury,” and the specific condition under discussion, tailoring the search to capture the most relevant studies. Notably, this review is not meant to be exhaustive of all biomarkers that predict patient outcomes. Our focus was on clinically relevant biomarkers that have been extensively studied and published, as well as occasionally outlining novel biomarkers with significant potential for addressing burn-induced conditions, despite being less studied. For novel biomarkers, we emphasized their promise while clearly stating their preliminary nature and the need for further validation. This approach allowed us to provide a balanced perspective on established biomarkers while highlighting emerging opportunities for advancing burn care. Additionally, this review highlights biomarkers originating from diverse tissues, including blood, serum, plasma, urine, burn tissue, and white adipose tissue.

### Biomarkers that Indicate severity, poor outcomes, and risk of mortality

**Fig. 1:**
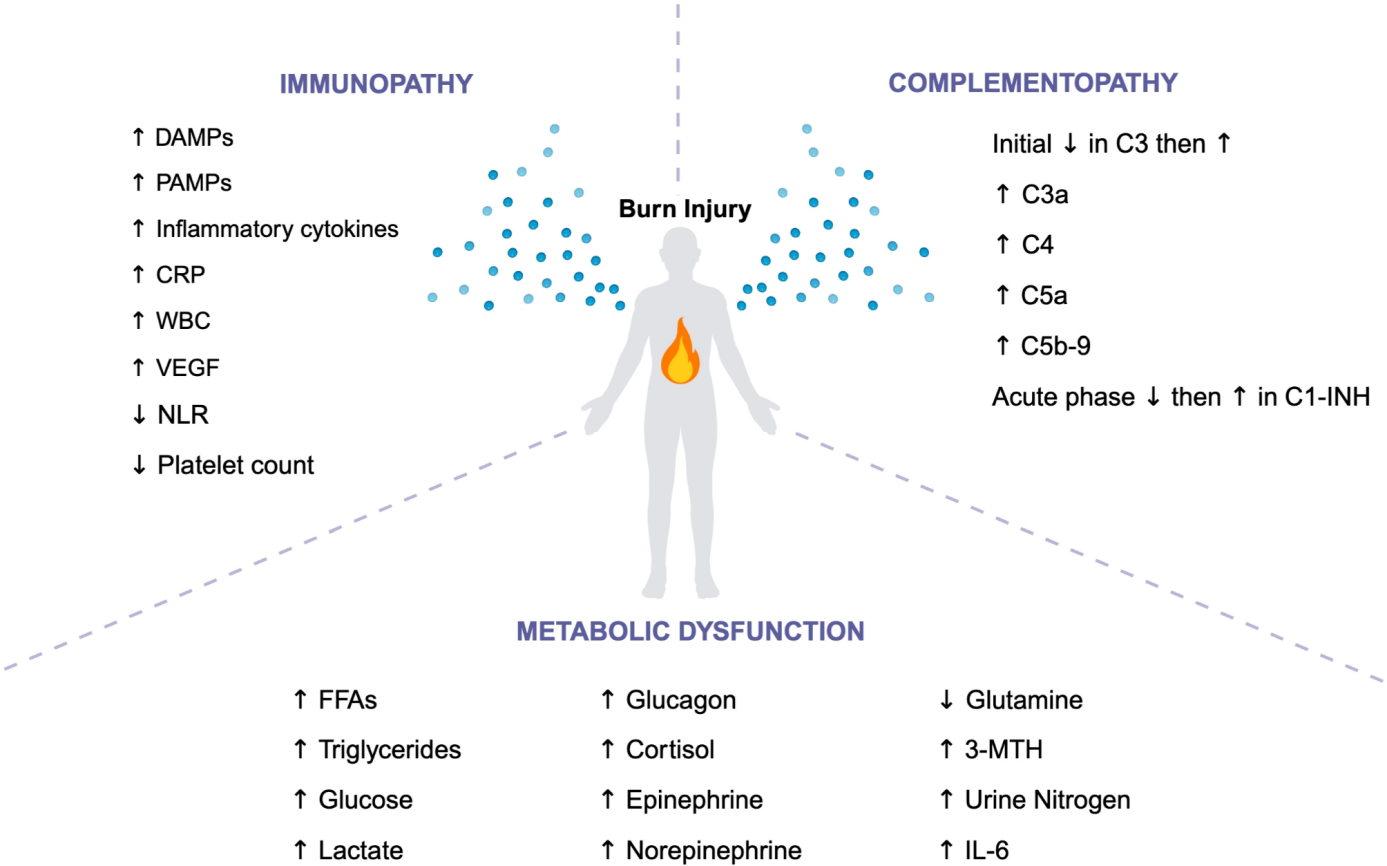
Changes in Levels of Biomarkers that Indicate Severity, Poor Outcomes, and Risk of Mortality in Burn Patients. A summary of the changes in levels of biomarkers that signify severity, poor outcomes, and risk of mortality post-burn, including immunopathy, complementopathy, and metabolic dysfunction. DAMPs, Damage-associated molecular pattern; PAMPs, Pathogen-associated molecular pattern; CRP, C-reactive protein; WBC, White blood cell; VEGF, Vascular endothelial growth factor; NLR, Neutrophil-lymphocyte ratio; C1-INH, C1 inhibitor; FFAs, free fatty acids; 3-MTH, 3-Methylhisidine; IL-6, interleukin-6. Created in BioRender. Jeschke, M. (2024) https://BioRender.com/j50s293

Before discussing the complications that arise from burn injuries, it is essential to first examine the burn-induced physiological alterations that precede and contribute to these complications, significantly influencing patient outcomes. These alterations include immunopathy, complementopathy, and metabolic dysfunction. The biomarkers associated with these conditions offer vital insights into the severity of the injury, the likelihood of adverse outcomes, and the overall risk of mortality (Fig. 1). Table 1 outlines selected biomarkers that indicate severity, poor outcomes, and risk of mortality in burn patients, with a particular focus on those that have been more extensively studied and consistently associated with these adverse outcomes.

**Table 1:** Biomarkers that indicate severity, poor outcomes, and risk of mortality

Biomarker	Function	Levels post-burn	Clinical implications
**Burn-Induced Immunopathy**			
Cytochrome C	Marker of mitochondrial damage (5,6)	Increased (5,6)	Indicates mitochondrial damage and correlates with burn injury severity (5,6)
HMGB1	Marker of cellular stress (5,6)	Increased (5,6)	Correlates with burn injury severity; indicates poor patient outcomes (5,6)
IL-6	Pro-inflammatory cytokine and central mediator in the acute phase response (7,8)	Increased (7,8)	Correlates with % TBSA burned, and depth of burn wound (7,8); associated with greater risk of mortality (7,9)
IL-8	Pro-inflammatory cytokine (7)	Increased (8)	Indicates future sepsis and correlates with burn severity (8)
IL-10	Anti-inflammatory cytokine (7,10)	Increased (7,10)	Increased levels predict sepsis and can distinguish survivors from non-survivors (10)
TNF-α	Pro-inflammatory cytokine (7)	Increased (11)	Correlates with poor outcomes and increased mortality risk (11)
IL-1β	Pro-inflammatory cytokine (7)	Increased (12)	Associated with greater risk of mortality (12)
IFN-γ	Pro-inflammatory cytokine (7,11)	Increased (7,11)	Associated with greater risk of mortality (7,11)
MCP-1	Pro-inflammatory cytokine (9)	Increased (9,13)	Correlates with burn severity and associated with a greater risk of mortality (9,13)
CRP	Acute inflammatory protein (7)	Increased (14)	High levels differentiate infection from other types of inflammation and predict infection risk (14)
Resistin	Adipokine and pro-inflammatory cytokine (15)	Increased (15)	Associated with greater risk of mortality (15)
NLR	Ratio of neutrophils to lymphocytes (16,17)	Decreased (16)	Higher ratio at admission associated with greater risk of mortality (16,17)
WBC Count	General marker of immune response (18)	Increased (18)	Increased WBC count shows positive correlation with length of hospital stay and mortality (18)
Platelet Count	Involved in clotting and wound healing (19,20)	Decreased (19)	Low platelet count associated with greater risk of mortality (19)
VEGF	Plays a role in angiogenesis and wound healing (21)	Increased (21)	Associated with greater risk of mortality; used to monitor wound healing progress (21)
**Burn-Induced Complementopathy**			
C3	Vital for complement activation and immune response (7,22,23)	Initially decreased, then increased (7,22,23)	Decreased C3 levels early post-burn associated with poor outcomes (7,23); later increases indicate prolonged inflammation and burn severity (22)
C3a	Involved in complement cascade (22)	Increased (22)	Correlates with trauma severity; associated with multiorgan dysfunction and increased mortality (22,24)
C4	Involved in complement cascade (7)	Increased (7,25)	Indicates prolonged systemic inflammation (7,25)
C5a	Involved in complement cascade (22)	Increased (24)	Greater risk of MODS and mortality (24)
C5b-9 (MAC)	Causes cell lysis and death (24)	Increased (24,26)	Associated with SIRS and MODS (24,26)
C1-INH	Inhibits activation of C1 complex and regulates complement activation (7)	Initially decreased, then increased (27)	Lower levels upon admission correlate with poorer outcomes (27); later increased levels suggest its potential as a prognostic marker (27)
**Metabolic Dysfunction**			
Free Fatty Acids	Key energy source (28)	Increased (28)	Contribute to fatty infiltration of vital organs (28)
Triglycerides	Key energy source (29–31)	Increased (29–31)	Elevated levels associated with MODS (29–31)
Glucose	Primary energy source (29)	Increased (30,32)	Indicates poor glucose control; hyperglycemia associated with higher mortality (30,32)
Lactate	Byproduct of anaerobic metabolism (29)	Increased (29)	Contribute to metabolic abnormalities and hyperglycemia (29)
Glucagon	Promotes glucose production in the liver during stress (29)	Increased (29)	Contributes to hyperglycemia (29)
Cortisol	Stress hormone (29)	Increased (29)	Increased glucose production and metabolic stress (29)
Epinephrine	Catecholamine (7,33)	Increased (8,34)	Elevated levels are associated with stress hypermetabolism, and inflammation (34)
Norepinephrine	Catecholamine (8,34)	Increased (8)	Elevated levels are associated with stress hypermetabolism, and inflammation (34)
Glutamine	Amino acid (35)	Decreased (35)	Is an energy substrate, decreased levels associate with hypermetabolism (35)
3-MTH	Amino acid (35)	Increased (35)	Correlates with muscle degradation and muscle protein loss (35)
Urine Nitrogen	Byproduct of protein metabolism (35)	Increased (35)	Increased levels correlate with protein catabolism (35)
IL-6	Also a biomarker of burn-induced immunopathy, refer to section above		

HMGB1: High mobility group box 1; IL: interleukin; IFN-γ: Interferon γ; MCP-1: Monocyte chemoattractant protein-1; CRP: C-reactive protein; NLR: Neutrophil-lymphocyte ratio; WBC: white blood cell; VEGF: Vascular endothelial growth factor; C1-INH: C1 inhibitor 3-MTH: 3-methylhistidine; TBSA: total body surface area; SIRS: systemic inflammatory response syndrome; MODS: multiorgan dysfunction syndrome

### Biomarkers of burn-induced immunopathy

Burn-induced immunopathy is a critical condition caused by severe burn injuries and significantly affects the functionality of the immune system, leading to an increased susceptibility to other post-burn complications. Following burn trauma, wound healing of the skin begins with an inflammatory stage to restore hemostasis and eliminate invading pathogens [5]. This inflammatory phase is initiated when damaged skin cells become necrotic or infected with pathogens, releasing damage-associated molecular patterns (DAMPs) or pathogen-associated molecular patterns (PAMPs), respectively [4, 5]. Once released, these DAMPs and PAMPs amplify the immune system and activate the acute phase response (APR) [4, 5].

Interestingly, promising animal research suggests that DAMPs, such as cytochrome C and high mobility group box 1 (HMGB1), may serve as potential biomarkers for immunopathy in burn patients, with evidence indicating their involvement across multiple tissues. Indeed, studies have demonstrated a positive correlation between burn injury size and circulating DAMP levels in a murine model [5, 6]. Moreover, cytochrome C, a marker of mitochondrial damage, is elevated eight-fold in mouse serum as early as three hours post-burn and remains elevated at 24 h, while HMGB1, shows a ten-fold increase in mouse serum at 24 h post-injury [6]. However, their application in human burn patients remains limited, as there is minimal clinical research exploring their utility in this context. While preclinical findings are encouraging, further research is necessary to validate these biomarkers and clarify their potential role in burn patient management. Until then, their clinical utility remains uncertain.

One of the primary responses to burn injury is the release of inflammatory cytokines including interleukin-6 (IL-6), interleukin-8 (IL-8), interleukin-10 (IL-10), interleukin-1β (IL-1β), tumor necrosis factor-α (TNF-α), interferon γ (IFN-γ), and monocyte chemoattractant protein-1 (MCP-1). Notably, IL-6 is significantly increased one to four days post-burn and plays a key role by stimulating acute phase protein (APP) synthesis in the liver, inducing naïve T-cell differentiation, and promoting angiogenesis [7, 8]. Indeed, in a study of 60 adult burn patients with total body surface area (TBSA) of 8–80%, all patients presented with significantly higher serum levels of circulating IL-6 within three days of burn injury [10]. However, IL-6 levels are not confined to the acute phase; rather, they remain significantly elevated for up to a month or even years after a burn injury [7, 8]. This elevation also correlates with the percent TBSA and depth of the burn wound [7, 8]. Additionally, studies have shown that on the day of burn trauma, plasma levels of IL-6 are significantly higher in non-survivors than in survivors, highlighting the reliability of IL-6 as a biomarker for predicting mortality [7, 9].

Moreover, IL-8—released by epithelial cells, endothelial cells, and macrophages—is important for neutrophil recruitment and tissue remodeling [7]. In a study performed by Jeschke et al., burn patients had dramatically elevated serum levels of IL-8 compared to healthy controls, with a 2000-fold increase being observed [8]. Similarly, IL-10 serum levels were found to peak on the first day post-burn and decline thereafter, with greatest concentrations correlating to percent TBSA burned and the presence of sepsis [7, 10]. Higher IL-10 serum levels are observed in septic versus non-septic patients, with levels of 60 pg/ml showing high sensitivity and specificity for distinguishing survivors from non-survivors [10]. IL-10 reaches its highest level around day three post-burn in septic non-survivors (180 pg/ml), while in non-septic and surviving septic patients, they remain relatively low at all times [10].

Inflammatory cytokines IL-1β and TNF-α surge during the influx of immune cells to the burn site, and their local persistence can last for weeks [7, 11, 12]. TNF-α—primarily produced by macrophages, neutrophils, and mast cells—plays a crucial role in the early systemic response post-burn by recruiting monocytes from the bloodstream [7]. A study by Yeh et al. found that an initial peak in serum TNF-α levels could be detected within 2.5 days after burn injury [36]. Further studies found that serum TNF-α levels have been observed to increase approximately four-fold in burn patients compared to healthy controls [11]. IL-1β, on the other hand, is a key pro-inflammatory mediator that is predominantly elevated not only at the burn site, but also systemically post-injury [7, 12]. In a study by Csontos et al. IL-1β expression in the blood was observed to reach its highest level on the third day post-injury in burns covering more than 20% TBSA [12]. Another inflammatory cytokine that can be observed as a marker of burn-induced immunopathy is IFN-γ. This marker is involved in the innate immune response and has been shown to be elevated four-fold in the blood of burn patients compared to healthy controls [11]. MCP-1 is another critical cytokine that plays a key role in recruiting monocytes to sites of tissue injury and has been shown to correlate with burn severity [13, 37, 38]. In a prospective observational study of 38 patients with ≥ 20% TBSA burns and 12 healthy controls, blood levels of MCP-1 were significantly higher in non-survivors than in survivors on day one post-burn [8]. While the small sample size limited statistical significance, blood MCP-1 levels on day two were still higher in non-survivors compared to survivors and controls [8]. Additionally, MCP-1 levels remained elevated in burn patients compared to controls between days three and five post-burn [8]. A study of severely burned pediatric patients also revealed that plasma MCP-1 levels, along with IL-6, and IL-8, dramatically increased within 24–48 h of trauma, correlating with injury size [8].

In response to a burn injury, alongside the release of inflammatory cytokines, PAMPs, and DAMPs, C-reactive protein (CRP) is rapidly released to trigger inflammation and activate the complement cascade (discussed in the next section), and remains elevated for months [7, 14]. In a study conducted by Jeschke and colleagues of 918 pediatric patients with an average TBSA of 45 ± 23%, plasma CRP levels significantly correlated with burn size, survival, and gender, as they were found at higher levels in large burns, amongst non-survivors, and in females [14]. In another study of adult burn patients, plasma CRP concentrations exceeding 8 mg/dl could distinguish infection-induced inflammatory responses from other types of inflammation, indicating its potential as a predictor of infection [39]. Massive burns, characterized by a TBSA greater than 80%, show the highest plasma CRP levels both acutely and up to six months post-burn, with significant elevation starting eight to ten days post-injury and persistently high levels beginning two to seven days post-burn [14].

In the acute phase of burns, resistin has also been implicated in burn severity and prognosis [15]. In a prospective observational study of 38 patients, blood resistin levels were significantly higher in non-survivors compared to survivors on the first day post-burn [15]. These findings suggest the potential of resistin as a biomarker for severity and mortality in major burns. However, research on this biomarker is limited, and further studies are required to confirm these findings. Additionally, neutrophil-lymphocyte ratio (NLR) is another emerging biomarker in burn patients. A study by Hu et al. of 271 patients with a median TBSA of 55%, found that NLR declined within the first three days after admission [16]. A high admission NLR, specifically above a ratio of 14, was negatively correlated with survival, suggesting that higher NLR may indicate poorer prognosis in burn patients [16]. Similarly, a study of 245 burn patients with ≥ 20% TBSA found that NLR was significantly higher in the mortality group compared to survivors, particularly on the seventh day post-burn [17]. At this time point, NLR was independently associated with mortality, with a sensitivity of 75% and specificity of 83.43% [17].

Lastly, white blood cell (WBC) count also serves as a marker of immunopathy, with studies showing that WBC counts are significantly higher in non-survivors compared to survivors within 48 h of injury [18]. In an observational study of 35 burn patients, WBC count showed a positive correlation with the length of hospital stay and TBSA, and peaked around 24–48 h [18]. Although WBC counts can fluctuate over time, with some reports noting decreases around two to five days post-burn followed by subsequent rises, WBC is particularly informative as a biomarker during the initial stages of injury [18, 40]. Similarly, in a study by Gajbhiye and colleagues of 594 adult burn patients, survivors demonstrated a gradual rise in blood platelet count, with 86.09% of survivors maintaining normal platelet levels before discharge [19, 20]. Conversely, non-survivors exhibited a gradual decline in blood platelet count, with 62.11% showing low platelet counts before death [19, 20]. Finally, vascular endothelial growth factor (VEGF) serum levels have been found to be significantly elevated post-burn, peaking at day 14 with a 22-fold increase compared to healthy controls and returning to normal after wound closure [21].

The biomarkers of immunopathy highlight the complexity of burn-induced immune dysregulation and the challenges of translating these insights into clinical practice. While biomarkers like DAMPs show promise in animal models, they remain underexplored in humans, emphasizing the need for further translational research. Additionally, cytokines fluctuate dynamically in response to various physiological and pathological processes, making them unreliable as independent or sole biomarkers for assessing burn-induced immunopathy and predicting outcomes. In fact, given the multifaceted nature of immunopathy, it is most likely that no single biomarker will suffice for diagnosis. Instead, a comprehensive approach using multiple biomarkers is necessary to both identify immunopathy and understand its underlying mechanisms. That being said, NLR has been independently shown to predict mortality, highlighting its potential as a reliable biomarker of immunopathy and survival. However, additional clinical research is needed to confirm this conclusion. Despite current diagnostic limitations, ongoing research into these biomarkers could reveal critical insights into how immunopathy contributes to the complications seen in burn patients. Having discussed the immune dysregulation caused by burn injuries, we now turn our focus to the complement system, another key player in the inflammatory response, whose dysfunction—referred to as complementopathy—further exacerbates burn-induced pathologies.

### Biomarkers of burn-induced complementopathy

The complement system plays a crucial role in the body’s innate immune response, inflammation regulation, pathogen defense, and maintenance of homeostasis [7, 22, 24]. Burn-induced complementopathy involves the hyperactivation of the complement system, marked by changes in regulation of various complement components [7, 22, 24]. Key biomarkers indicating the activation and regulation of the complement system post-burn injury include complement factors C3, C3a, C4, C5a, C5b-9, and C1 esterase inhibitor (C1-INH). However, diagnosing complementopathy has proven to be challenging because the active levels of complement pathway proteins heavily depend on age and storage factors, making it difficult to quantify the normal range of complement factors [22].

Complement factor C3 is one of the most abundant plasma proteins and plays a vital role in complement activation [41]. C3 is typically present at a high plasma concentration of around 1.2 mg/mL [41]. Interestingly, da Silva et al. found that plasma C3 concentrations as low as 0.18 mg/mL are sufficient to maintain proper activation of complement response and prevent complement-related diseases [41, 42]. Following severe burn injury, studies have reported an initial decrease in serum C3, possibly due to increased permeability of local blood vessels, increased APP turnover rate, and a decrease in APP production [7, 43]. However, C3 begins to continuously increase a few days post-burn, reaching a plateau on day seven, and potentially remaining elevated for months [7, 22, 23]. Intriguingly, a study by Modi et al. reported an inverse correlation between blood C3 levels and burn severity, highlighting its potential as a prognostic marker [22, 44]. Moreover, C3a, which is derived from the cleavage of C3, induces inflammation by attracting immune cells to the site of injury [22]. Post-burn, plasma C3a levels increase rapidly and correlate directly with the severity of the trauma [22, 24]. Similar to C3, plasma C3a concentrations peak approximately one-week post-burn [22, 24]. Notably, elevated plasma C3a levels have been associated with the development of multiorgan dysfunction syndrome (MODS) and increased mortality [22, 24]. Interestingly, complement factor C4, although similarly elevated following trauma, shows a distinct pattern compared to C3 [7, 25]. Studies conducted in a pig burn wound model have shown that blood C4 levels rise for a shorter duration and peak later than C3 [7, 25]. Despite normalization of local C3 and C4 levels at the burn wound site, plasma concentrations remain elevated, suggesting a prolonged systemic inflammatory response mediated by the complement system [7, 23, 25]. However, research investigating C4 as a biomarker in human burn patients remains limited, and further studies are necessary to clarify its role and clinical utility in burn patient management.

Similar to C3, complement factor C5 is cleaved into C5a and C5b, which attract immune cells and neutrophils to the site of injury, contributing to inflammation and tissue damage [22]. Elevated plasma C5a levels are indicative of complement activation and have been linked to increased inflammation and injury severity in burn patients [24]. Several studies have reported an increase in plasma concentrations of C5a following burn injury [24]. Although, it has been determined that C5a directly correlates with percent TBSA, MODS, and mortality, the literature is controversial with regards to when the highest C5a concentrations are observed [22, 24]. Finally, the last stage of complement activation is the formation of the C5b-9 complex, which leads to cell lysis and death [22]. Studies have shown that serum C5b-9 concentrations in trauma patients are significantly higher than in healthy individuals, with notable increases by day two post-injury, which remained more than two-fold higher than levels at admission through day seven [24]. Additionally, a study involving 33 trauma patients found that elevated serum C5b-9 levels were positively associated with the occurrence of systemic inflammatory response syndrome (SIRS) [24]. Although most studies focus on general trauma, specific correlations have been made with burn injuries, where both burn and blunt trauma patients exhibit higher serum C5b-9 concentrations than those with penetrating injuries [24]. In humans, enhanced C3a, C5a, and C5b-9 concentrations in the blood have been proposed as a driver for sepsis-induced complications and MOF [22, 26].

Lastly, C1-INH is a crucial regulatory protein in the complement system and is primarily responsible for inhibiting the activation of the C1 complex [7]. In a study of 38 patients with burns ≥ 20% TBSA, plasma C1-INH activity was found to acutely decrease for the first 48 h, followed by a gradual increase above reference levels from days three to five, after which it continued to rise [27]. Further studies revealed lower plasma C1-INH activity upon admission is significantly correlated with poorer outcomes and mortality [27]. Matsuura and colleagues similarly found that C1-INH activity on admission was significantly lower in non-survivors (59% activity) than in survivors (90% activity) during a 28-day evaluation period [27]. These findings underscore the potential of C1-INH as a prognostic marker for burn patients and highlight the importance of early intervention to modulate its activity.

Current diagnostic practices for complementopathy lack specificity and primarily rely on general inflammatory markers, making it difficult to precisely diagnose complement dysfunction in burn patients. The biomarkers of complementopathy, such as C3, are central to understanding this condition, as C3 plays a key role in complement activation and is the most studied biomarker. C3 levels have shown relatively consistent patterns among burn patients, offering a potential diagnostic tool. However, biomarkers like C4 remain underexplored in humans, limiting their clinical application. While these biomarkers show promise, no single one is sufficient on its own, and a combination of biomarkers is needed to better evaluate complementopathy. As complementopathy is often grouped with broader immunopathy, further research is needed to improve diagnostic criteria. Now that we have explored the inflammatory response to burn injuries, we will shift our focus to the resulting metabolic dysfunction, which arises as a consequence of this prolonged immune activation and further contributes to systemic damage.

### Biomarkers of burn-induced metabolic dysfunction

Metabolic dysfunction refers to a range of abnormalities in the body’s metabolism. Following burn trauma, two distinct sequences of metabolic regulation can be observed [29]. The first phase, early shock metabolism (ebb phase), typically occurs within the first 48 h post-burn [29, 30, 45]. This immediate response presents with decreased cardiac output, lower oxygen consumption, reduced metabolic rate, and hyperglycemia [29, 30, 45]. The second phase, known as the flow phase, begins within the first five days post-burn, where metabolic activity gradually increases [29, 30, 45]. This plateau phase is characterized by hyperdynamic circulation, leading to elevated body temperature, increased oxygen and glucose consumption, higher CO_2_ production, and futile substrate cycling [29, 30, 45]. The flow phase induces a severe state of hypermetabolism that can persist for years after the initial burn injury, resulting in a range of harmful downstream consequences [30]. These downstream consequences are often characterized by biomarkers that indicate the presence of metabolic alterations.

As previously discussed, indicators such as pro-inflammatory cytokines (e.g., TNF-α, IL-6) and catecholamines (e.g., epinephrine and norepinephrine) are also closely linked with hypermetabolism [29, 30, 45]. These inflammatory mediators are typically elevated post-burn, with IL-6 being one of the first cytokines to be detected in plasma and is significantly and consistently associated with the hypermetabolic response [46]. IL-6 has been shown to correlate with the elevated resting energy expenditure (REE) observed in burn patients, which is indicative of increased metabolic demand and hypermetabolism [28, 47]. Interestingly, in a study conducted by Abdullahi et al., the adipose tissue of burn patients also exhibited significantly elevated IL-6 levels compared to healthy controls, further corroborating its role in the post-burn hypermetabolic response [47]. Unsurprisingly, catecholamines, also tend to be elevated alongside REE following burns [8, 34]. In fact, it is well established that catecholamines drive the hypermetabolic response to thermal injury in adults, a phenomenon that has also been confirmed in pediatric burn patients [43, 48]. Considering the well-established role of catecholamines in mediating the hypermetabolic response, we will not delve into their mechanisms in detail in this review. Notably, hypermetabolism in burn patients is also associated with altered glucose, lipid and protein metabolism, making biomarkers critical for tracking these changes.

In fact, elevated lipolysis rates are observed in both humans and animals following a burn injury, resulting in increased levels of free fatty acids (FFAs) which appear immediately after the injury and can persist anywhere from five days to two months [28, 49, 50]. Stanojcic et al. analyzed the clinical outcomes of 1,288 adult burn patients over a 10-year period, with data collected from white adipose tissue (WAT), serum, and plasma [51]. During the first 14 days post-burn, increases in pro-inflammatory fatty acids, particularly stearic acid and linoleic acid, were observed [51]. Anti-inflammatory FFA levels demonstrated similar results, although their increase was less significant compared to the pro-inflammatory FFAs [51]. Consequently, the increased triglycerides (TG) and FFAs contribute to the fatty infiltration of crucial metabolically active organs, especially the liver [28]. Indeed, a recent clinical analysis in pediatric patients by Kraft et al. further confirms increased FFAs post-burn and reveals that elevated TGs are linked to deteriorating organ function and poorer clinical outcomes, highlighting the impact of these hypermetabolic responses in post-burn patients [29–31].

Moreover, when the body enters a hypermetabolic state post-burn, energy demands drastically increase, raising glucose levels [29]. As a result, the body releases stress mediators such as glucagon and cortisol which oppose the anabolic effects of insulin [29]. This triggers hepatic gluconeogenesis, increasing the production of glucose, promoting lipolysis and driving muscle proteolysis to provide the body with gluconeogenic and glycolytic substrates like glycerol, alanine, and lactate [29]. As glucose and lactate levels rise, they contribute to metabolic abnormalities such as hyperglycemia, profound catabolism, increased ectopic fat deposition, and wasted energy [29, 52]. Daily glucose monitoring by Stanojcic et al. revealed hyperglycemic and hyperinsulinemic responses that peaked during the first 7 to 14 days post-burn [51]. Hyperglycemia represents a clinical problem in burn patients, with several studies demonstrating higher mortality in burn patients with poor glucose control due to factors like impaired tissue extraction of glucose [30, 32]. This was demonstrated in a study conducted by Holm and colleagues which evaluated blood glucose levels to determine if maintaining these levels in patients with hyperglycemia would improve survival rates [53]. In fact, they observed that patients who did not survive had significantly higher glucose levels than those who did and by maintaining levels between 180 and 200 mg/dl, the survival rates increased [53]. Therefore, elevated glucose, lactate, glucagon, and cortisol levels should be closely monitored in burn patients.

Additionally, following burn injury, skeletal muscle serves as a primary source of fuel, with amino acids and proteins being rapidly metabolized [29, 30]. This accelerated protein breakdown leads to muscle wasting and a significant loss of lean body mass (LBM) within days post-burn [29, 30]. In fact, a study by Peng et al. found that both plasma glutamine and protein content were lower in burn patients, while urine nitrogen and 3-methylhisidine (3-MTH) significantly increased [35]. Although the production of the amino acid glutamine is elevated, its increased uptake by various organs and systems surpasses its synthesis, ultimately resulting in decreased glutamine levels [35]. Moreover, 3-MTH is an amino acid found in urine that serves as a marker for muscle catabolism. Its elevated levels post-burn indicates increased muscle degradation, making it valuable for identifying muscle protein loss [35]. Indeed, in a study evaluating amino acid levels in exudate collected from adult burn patients, researchers found that the greatest amino acid loss occurred during the first three days, with glutamine undergoing the most significant depletion [54]. Similarly, Biolo et al. performed a study to determine levels of amino acids from blood samples drawn from muscle tissue [55]. Their findings also revealed a decrease in glutamine concentrations alongside increased alanine concentrations [55].

The post-burn hypermetabolic response is not only intense but also notably prolonged, with evidence suggesting it can persist for up to five years after the initial injury [8]. This response evolves dynamically through distinct phases, driven by shifting metabolic demands, making it difficult to identify biomarkers that are both specific and sensitive. Substrates involved in resource catabolism—such as amino acids, glucose, and FFAs—are unreliable as consistent markers of hypermetabolism, as their breakdown occurs under varying conditions and is not always synchronized. However, while these biomarkers are limited in their diagnostic capacity, over a longitudinal period, they remain essential for understanding the phases of hypermetabolism and how metabolic priorities change over time. REE can help confirm the presence of hypermetabolism, aiding diagnosis, but does not capture the specific needs of individual patients. These biomarkers are crucial for determining when hypermetabolism serves an adaptive purpose and when it becomes detrimental, leading to excessive tissue breakdown or immune dysfunction. This understanding can guide timely, targeted interventions to address the metabolic demands of burn patients more effectively. Ultimately, the development of reliable biomarkers will be key to unraveling the physiological mechanisms that transition hypermetabolism from a necessary acute response to a chronic, harmful state, allowing for more precise therapeutic strategies. After considering the physiological alterations that can indicate severity, poor outcomes and mortality, we will delve into the pathophysiological conditions and complications that arise from these alterations.

### Biomarkers of burn-induced pathophysiological conditions and complications

**Fig. 2:**
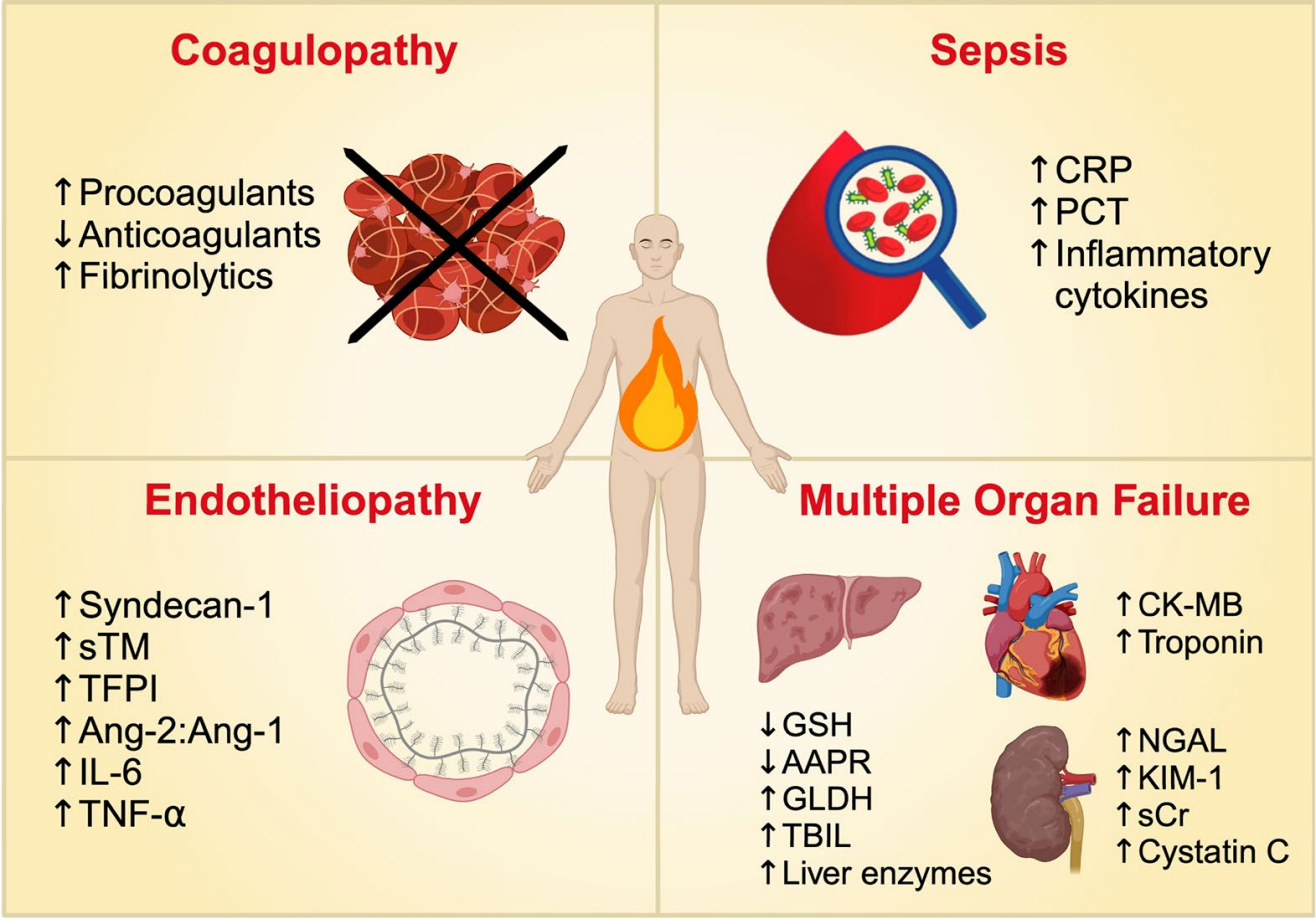
Overview of Biomarker Alterations in Post-burn Pathophysiological Conditions and Complications. A summary of the changes in levels of biomarkers as a result of post-burn pathophysiological conditions and complications, including coagulopathy, sepsis, endotheliopathy, and multiple organ failure. CRP, C-reactive protein; PCT, Procalcitonin; sTM, Soluble thrombomodulin; TFPI, Tissue factor pathway inhibitor; Ang-1, Angiopoietin-1; Ang-2, Angiopoietin-2; IL-6, interleukin-6; TNF-⍺, tumor necrosis factor-⍺; GSH, Glutathione; AAPR, Albumin-to-alkaline phosphatase ratio, GLDH, Glutamate dehydrogenase; TBIL, Total bilirubin; CK-MB, Creatine kinase-MB; NGAL, Neutrophil gelatinase associated lipocalin; KIM-1, Kidney injury molecule-1; sCR, Serum creatinine. Created in BioRender. Jeschke, M. (2024) https://BioRender.com/j50s293

Immunopathy, complementopathy, and metabolic dysfunction following a burn injury disrupt the body’s homeostasis, leading to severe complications and physiological conditions. These states of dysfunction are typically correlated with the size and severity of the burn—worsening as the extent of the injury increases, which, in turn, exacerbates the resulting complications and pathological conditions. In this section, we will explore these burn-induced conditions leading to poor outcomes and examine potential biomarkers that can facilitate earlier diagnosis and intervention. These conditions include: endotheliopathy, coagulopathy, multi-organ failure, and sepsis (Fig. 2). Table 2 highlights the biomarkers that are extensively studied for these burn-induced complications and pathophysiological consequences.

**Table 2:** Biomarkers of burn-induced pathophysiological conditions and complications

Biomarker	Function	Levels post-burn	Clinical implications or signified outcomes
**Endotheliopathy**			
Syndecan-1	Major component of the endothelial glycocalyx (33)	Increased (33,56,59)	Poor patient outcomes including mortality (33,56,59)
sTM	Cleaved form of thrombomodulin (59)	Increased (59)	Independent predictor of 7-day and 28-day mortality (60)
TFPI	Coagulation inhibitor (56)	Increased (56)	Greater mortality risk; independent predictor of 30-day in-hospital mortality (56)
Ang-2:Ang-1	Growth factors and Tie-2 receptor antagonist (Ang-2) and agonist (Ang-1) (61)	Increased (61,62)	Higher ratio found amongst those with greater disease burden as well as in non-survivors (62)
IL-6, TNF-⍺	Also biomarkers of burn-induced immunopathy, refer to Table 1		
**Coagulopathy**			
VIIa	Activated form of factor VII (80)	Increased (80)	Higher levels present in non-survivors (80)
VIIc	Procoagulant factor (80)	Low (80)	Lower post-burn day 1 levels in survivors (80)
TAT Complex	Marker of thrombin generation and neutralization (65)	Increased (65,66,80,81)	Day 7 TAT levels are prognostic indicator for ICU mortality (65,81)
F1.2	Marker of thrombin generation (65)	Increased (65,81)	Reflects increased thrombin generation (65)
Antithrombin	Natural anticoagulant (65,80,81)	Decreased (65,80,81)	Decreased antithrombin represents greater thrombogenicity (65); low antithrombin levels predicts mortality and length of hospital stay (64,82)
PC	Natural anticoagulant (65,80,81)	Decreased (65,80,81)	Days 5 and 7 PC levels serve as independent predictors of ICU mortality (81)
PS	Natural anticoagulant (65,81)	Decreased (65,81)	Day 3, 5, and 7 PS levels are independent predictors of ICU mortality (81)
t-PA	Factor of the fibrinolytic system (65)	Increased (64–66,80,81)	Associated with disseminated intravascular coagulopathy (80)
PAI-1	Factor of the fibrinolytic system; inhibits t-PA (65,80)	Increased (65,66,80,81)	PAI-1 counterbalances effects of t-PA (65); day 3 PAI-1 levels offer prognostic value for ICU mortality (65)
D-dimer	Fibrin degradation product (65)	Increased (65,80,81)	Indicates greater thrombin generation, fibrin formation and degradation (65)
**Hepatic Dysfunction**			
ALT and AST	Liver enzymes (85–87,89)	Increased (85–87,89)	Indicators of hepatocyte damage; correlated with extent of liver injury (85–87,89)
ALKP	Serum enzyme related to liver function (7)	Increased (43,85,86)	Elevated levels indicate extent of liver injury (43,85,86)
AAPR	Ratio of albumin to alkaline phosphatase (89)	Decreased (89)	Higher ratios associated with increased chance of ICU discharge (89)
GLDH	Mitochondrial enzyme found in the liver (43)	Increased (7,90,91)	Elevated levels indicate extent of liver injury (7,90,91)
TBIL	Waste product processed by liver (88)	Increased (88)	Indicative of liver dysfunction; associated with increased mortality (88)
GSH	Protects hepatocytes from oxidative stress (92)	Decreased (92)	Increased oxidative stress and hepatocyte damage (92)
**Renal Failure**			
sCR	Waste product filtered by kidneys (100)	Increased (100)	Indication of kidney damage and decreased renal function (100)
Cystatin C	Involved in kidney filtration (100)	Increased (100)	Poor kidney function (100)
NGAL	Associated with kidney injury (100)	Increased (100)	Risk of renal complications and mortality (100)
KIM-1	Transmembrane protein (101)	Increased (101)	Associated with early indication of kidney injury (101)
**Cardiac Dysfunction**			
CK-MB Isoenzyme	Isoenzyme specific to cardiac muscle (102,107)	Increased (102,107)	Indication of heart damage (102,107)
cTnI	Regulatory protein specific to cardiac muscle (95,100,101)	Increased (95,100,101)	Elevated concentrations are found only in presence of cardiac injury (95,100,101)
**Sepsis**			
CRP	Acute inflammatory protein (131)	Increase (2,113,114)	Predictor of infection; can indicate sepsis before appearance of clinical symptoms (113,114)
PCT	Precursor of the calcitonin hormone (2,116)	Increased (116–118)	Lower levels found in surviving septic patients (111,118,119,121)
IL-6, IL-8, IL-10, IFN-ɣ, TNF-⍺	Also biomarkers of burn-induced immunopathy, refer to Table 1		

sTM: Soluble thrombomodulin; TFPI: Tissue factor pathway inhibitor; Ang: Angiopoietin; VIIa: Activated factor VII; VIIc: Procoagulant factor VII; TAT: Thrombin-antithrombin; F1.2: Prothrombin fragment; PC: Protein C; PS: Protein S; t-PA: Tissue plasminogen activator; PAI-1: Plasminogen activator inhibitor 1; ALT: Alanine transferase; AST: Aspartate aminotransferase; ALKP: Alkaline phosphatase; AAPR: Albumin-to-alkaline phosphatase ratio; GLDH: Glutamate dehydrogenase; TBIL: Total bilirubin; GSH: Glutathione; sCR: Serum creatinine; NGAL: Neutrophil elatinase associated lipocalin; KIM-1: Kidney injury molecule-1; CK-MB: Creatine kinase-MB; cTnI: Cardiac troponin I; CRP: C-reactive protein; PCT: Procalcitonin

### Biomarkers of endotheliopathy

The hyperinflammatory state that arises following a burn injury increases metabolism, activates the endothelium, and damages the endothelial glycocalyx layer [2]. The endothelial glycocalyx consists of a network of proteoglycans and glycoproteins on the luminal surface of blood vessels [33, 56]. The integrity of this layer is critical for homeostasis as it protects the endothelium, maintains vascular barrier function, and regulates inflammation, blood clotting, and blood flow [2, 56]. Endothelial activation and disruption of the endothelial glycocalyx layer, termed traumatic endotheliopathy, are key drivers of downstream systemic effects [33]. Therefore, using biomarkers to recognize and assess the extent of endotheliopathy experienced by burn patients could be useful to clinicians, providing them with prognostic information to help guide decision-making.

During the trauma-induced hyperinflammatory response, elevated catecholamine levels lead to the disruption of the glycocalyx, and consequently the shedding of syndecan-1 [33]. Syndecan-1 is a glycoprotein and a major component of the endothelial glycocalyx [33]. In fact, increases in serum syndecan-1 concentrations are proportional to the extent of endothelial glycocalyx damage [57]. Further, median admission plasma syndecan-1 levels have been associated with burn size, increasing from small (15.7 ng/mL), to moderate (25.7 ng/mL), to large (37.6 ng/mL) burns [56]. These findings suggest that the extent of endothelial dysfunction post-burn is burn size-dependent and can be assessed as early as upon hospital admission, allowing for prompt and focused treatment of endotheliopathy, thereby enhancing patient outcomes [56]. Interestingly, Welling et al. found that the extent of endothelial glycocalyx shedding is associated with the presence of inhalation injury rather than burn size [33]. Similarly, Osuka and colleagues reported that serum levels of syndecan-1 are correlated with age, but not with burn size [58]. Thus, further research must be carried out to confirm these findings. Various researchers have identified cutoffs for this biomarker which indicate poor patient outcomes. In a prospective observational study of burn patients, Welling et al. found that endotheliopathy of trauma, characterized by plasma syndecan-1 levels of 40 ng/mL, are associated with higher morbidity and mortality [33]. This cutoff has also been found to identify trauma patients with significantly poorer outcomes, in the absence of clinically significant differences in admission physiology [59]. Interestingly, Keyloun and colleagues found that a lower threshold of plasma syndecan-1 levels, specifically those exceeding 34 ng/mL, were linked to a 32-fold increase in risk of mortality and a 14-fold decrease in time until death [56]. Higher admission syndecan-1 levels have been correlated with higher risk of mortality and showed comparable performance to the revised Baux score in predicting 30-day in-hospital mortality, suggesting that plasma syndecan-1 levels are directly linked to burn patient injury severity [56]. Overall, syndecan-1 serves not only as a main biomarker of endotheliopathy, but also as a quantitative measure for the condition, offering an objective measure of endothelial glycocalyx breakdown and endothelial damage that can be used to evaluate the progression of this syndrome and guide clinical decisions [59].

Another biomarker of endotheliopathy is thrombomodulin, an anticoagulant protein found on the surface of endothelial cells which contributes to activating the protein C anticoagulant pathway [59]. Following trauma, the rise of inflammatory cytokines TNF-α and IL-6 lead to the downregulation of thrombomodulin [59]. Neutrophils are also activated, which cleave thrombomodulin, shedding it into the bloodstream as soluble thrombomodulin (sTM), a well-recognized biomarker of endothelial cell damage [59]. In a prospective observational study of trauma patients, plasma levels of sTM in patients with endotheliopathy were found to be approximately 1.5 times greater than those without endotheliopathy (median 6.7 ng/mL compared to 4.7 ng/mL, respectively) [59]. Additionally, researchers found a moderate positive correlation between plasma levels of sTM and syndecan-1 [59]. Interestingly, in a study of 424 trauma patients, sTM was found to be an independent predictor of 7-day and 28-day mortality, along with age [60].

Moreover, Tissue Factor Pathway Inhibitor, or TFPI, is a coagulation inhibitor mainly produced by endothelial cells and is released into the plasma as a biomarker of endothelial damage [56]. Similar to syndecan-1, median admission plasma levels of TFPI have been found to increase with burn size, progressing from small burns (64.4 ng/mL), to moderate-sized burns (72.4 ng/mL), to large burns (81.9 ng/mL) [56]. Furthermore, increased plasma TFPI levels at admission have been associated with a greater risk of mortality, with levels greater than 73 ng/mL associated with a nine-fold increased risk [56]. Additionally, TFPI was found to be an independent predictor of 30-day in-hospital mortality [56]. Although TFPI was found to be a fair predictor of mortality, syndecan-1 was more effective [56].

Lastly, angiopoietin-2 (Ang-2), a growth factor and Tie-2 antagonist, destabilizes blood vessels, increases vascular leakage, promotes vascular regression, and prepares the endothelium to respond to angiogenic and inflammatory cytokines [61]. In a study conducted by Ganter and colleagues, blood samples of 208 adult trauma patients were collected within a half hour of injury and prior to substantial fluid resuscitation [61]. The researchers found that plasma levels of Ang-2 were elevated proportional to the degree of injury severity and tissue hypoperfusion [61]. In contrast, levels of the Tie-2 receptor agonist, angiopoietin-1 (Ang-1), remained unchanged [61]. Given the agonist-antagonist nature of Ang-1 and Ang-2 on the endothelium, assessment of their ratio, rather than the absolute concentration of either ligand is suggested to better indicate endothelial activation [61]. In the same study, trauma patients who suffered severe injury or shock presented with elevated and unchanged plasma concentrations of Ang-2 and Ang-1, respectively– therefore, a low Ang-1:Ang-2 ratio, signifying activation of endothelial cells soon after injury [61]. Plasma levels of Ang-2 were further correlated with biomarkers of endothelial activation, abnormalities in coagulation, and increased complement cascade activation [61]. Elevated Ang-2 plasma concentrations were also associated with worse clinical outcomes [61]. These findings indicate that Ang-2 serves as a marker and a potential direct mediator of endothelial activation and dysfunction following severe trauma [61]. Notably, when investigated in burn patients, researchers found that the serum Ang-2: Ang-1 ratio rises during the first 48 h post-burn [62]. Moreover, this ratio is found to be higher in those who succumb to their injuries as well as those with greater disease burden in terms of abbreviated burn severity index and TBSA [62]. Further studies should be conducted to confirm the usefulness of this biomarker and its relation to endothelial activation and dysfunction in burn patients.

Syndecan-1 is often considered one of the most useful biomarkers of endotheliopathy in the context of burns, offering insight into the severity of vascular injury. It is the most studied biomarker in burn-related endothelial dysfunction, with research suggesting an association with both endothelial damage and the inflammatory response. However, unlike the Ang-2:Ang-1 ratio, syndecan-1 levels do not correlate strongly with TBSA burned, a key determinant of burn severity. The Ang-2:Ang-1 ratio, in contrast, appears to have a clearer connection to TBSA and may more directly reflect vascular instability. Combining these biomarkers could provide a more comprehensive assessment by accounting for different prognostic factors. Additionally, because endothelial dysfunction evolves dynamically over time, biomarkers alone may not fully capture the extent of injury or recovery. A panel of biomarkers could allow for a more accurate and longitudinal view of the patient’s condition, helping to track the progression of endothelial damage and repair.

### Biomarkers of coagulopathy

Burn patients, especially those who have sustained severe burn injuries, often exhibit changes in their coagulation system [63–65]. In fact, the profound systemic inflammatory response (SIR) triggered by thermal injury disrupts the balance between coagulation and fibrinolysis [65–68]. Although controlled activation of the coagulation system is crucial for wound healing, uncontrolled activation of coagulation factors can result in disseminated intravascular coagulation, microvascular thrombosis, hypoperfusion, MOF, and increased morbidity and mortality [65]. Development of microthrombosis disrupts the circulation in the wound, resulting in increased tissue necrosis [69]. This process contributes to the worsening and deepening of the wound after a burn injury [69]. During the acute phase post-burn, coagulopathy can be triggered by various factors, including tissue hypoperfusion, hypothermia, hemodilution, endothelial damage, or SIRS [63, 64, 70–72]. Moreover, burn-related complications, such as inhalation injury, sepsis and bleeding from excisional surgeries, can further contribute to changes in coagulation [64, 73, 74]. Notably, the extent of coagulopathy has been found to be proportional to TBSA burned, while both the onset and degree of hemostatic change correlate to the severity of the burn [63, 64, 72, 75, 76]. In fact, patients who have sustained severe burn injuries often exhibit or develop extensive coagulopathy, while those with mild to moderate burns usually do not [64, 65, 72, 75, 77–79]. Interestingly, coagulopathy is also an independent predictor of 28-day mortality in patients who have suffered severe burn injury [64, 65, 75]. However, despite its potential for being an effective predictor, the early identification of coagulopathy can be difficult [64]. Routine coagulation tests such as prothrombin time (PT) and activated partial thromboplastin time (APTT) are poor diagnostic tools for detecting coagulation abnormalities in burn patients [63, 64]. In fact, notable alterations in both coagulation and fibrinolytic markers have been observed in the acute phase post-burn despite normal PT and APTT, thereby highlighting the diagnostic potential of these biomarkers [79].

### *Procoagulant biomarkers*

Coagulopathy in burn patients is in part characterized by changes in procoagulant proteins [64]. Factor VII (VIIc) is a procoagulant factor that plays an important role in the coagulation cascade as it initiates the extrinsic coagulation pathway. On the first day post-burn, low levels of activity of VIIc have been observed [80]. Interestingly, on the seventh day, VIIc activity returns to near-normal in non-survivors, while remaining low in survivors [80]. Researchers have also investigated changes in levels of VIIa, the activated form of factor VII, which were found to be elevated in survivors and non-survivors on the first-day following burn injury, with non-survivors presenting with significantly higher levels [80]. Meanwhile, on the seventh day post-burn, levels of VIIa are observed to decrease and signal towards normality, yet they remain higher than those found in healthy individuals and are higher amongst non-survivors [80].

Another biomarker of coagulation is the thrombin-antithrombin complex, or TAT complex. On the first day post-burn, TAT levels are elevated above normal values in both survivors and non-survivors but decrease during the first week [65, 66, 80, 81]. Interestingly, survivors appear to have significantly lower values of TAT on the seventh day post-burn than non-survivors [65, 80, 81]. In fact, TAT levels on the seventh day were found to serve as a prognostic indicator for intensive care unit (ICU) mortality [65, 81]. Lastly, researchers have found that levels of procoagulant biomarker, prothrombin fragment 1.2 (F1.2), remain elevated during the first week post-burn [65, 81]. However, no significant differences in F1.2 levels have been observed between survivors and those who succumb to their injuries [65, 81]. Thus, while it may serve as an indicator of coagulopathy, it is not effective in predicting mortality, unlike TAT.

### *Anticoagulant biomar*kers

Coagulopathy in burn patients is also characterized by impairments to the natural anticoagulant systems [64]. During the acute post-burn response, levels of antithrombin, a coagulation inhibitor, decrease, but in some studies have been shown to normalize in survivors on the fifth day post-burn, while others report levels remaining low on the seventh day post-burn [65, 80, 81]. The decrease in antithrombin levels, together with the increase in TAT, reflect both increased consumption of antithrombin and greater thrombogenicity [65]. Notably, low antithrombin levels have been found to independently predict both mortality and length of hospital stay [64, 82]. In fact, in Lavrentieva and colleagues’ study, the researchers found day three antithrombin to have good prognostic value for ICU mortality as indicated by ROC AUC analysis [65]. Additionally, protein C (PC) serves as another key anticoagulant factor. Similar to antithrombin, levels of PC initially decrease but return to normal in survivors between days five and seven post-burn [65, 80, 81]. Interestingly, however, Garcia-Avello and colleagues found PC levels on the seventh post-burn day to remain low [80]. Results from a logistic regression analysis showed that PC at days five and seven serves as an independent predictor of ICU mortality [81]. Similarly, day five PC was found to have good prognostic value for ICU mortality as shown by ROC AUC analysis [65]. Lastly, the anticoagulant marker protein S (PS) exhibits a trend similar to that of antithrombin and PC after a burn. Its levels are initially decreased but return to normal in survivors between days five and seven [65, 81]. Likewise, PS was found to be an independent predictor of ICU mortality on days three, five, and seven based on logistic regression analysis [81]. ROC AUC analysis also showed that day three PS has good prognostic value for ICU mortality [65].

### *Fibrinolytic biomarkers*

Coagulopathy in victims of burn injury is lastly characterized by impaired fibrinolytic activity [64]. Tissue plasminogen activator, or t-PA, is one of many biomarkers of fibrinolysis. Levels of this biomarker have been found to be elevated as early as the day of the burn but exhibit significant decreases on days five to eight, although in some studies the levels remain above clinically normal values [64–66, 80, 81]. However, other researchers have found that t-PA levels normalize after day five in surviving patients [65]. Furthermore, plasminogen activator inhibitor 1 (PAI-1), which inhibits t-PA, also serves as a biomarker of fibrinolysis. PAI-1 levels have been found to be elevated in burn patients as early as admission, although differences exist in the literature with regards to the changes observed in the levels of this biomarker during the first week post-burn [65, 66, 80, 81]. While some researchers have observed consistently elevated levels of PAI-1 in burn patients between days five and eight, others have reported a significant decrease in PAI-1 levels on day seven compared to admission levels in survivors, and some have found that PAI-1 levels normalize at day five in surviving patients [65, 66, 80, 81]. Thus, further research must be conducted to confirm these findings. Interestingly, using ROC AUC analysis, Lavrentieva and colleagues found PAI-1 on day three to have good prognostic value for ICU mortality [65]. Lastly, D-dimer (DD), another fibrinolytic marker, has been shown to be elevated above normal values on the first day post-burn in patients with greater than 20% TBSA, and remains elevated during the first week [65, 66, 80]. Interestingly, the rise in plasma levels of F1.2 and DD indicate greater thrombin generation, as well as enhanced fibrin formation and its subsequent breakdown [65]. Moreover, in a study conducted by Garcia-Avello and colleagues, the researchers found a significant difference in certain hemostatic markers on the first post-burn day between patients who had suffered burns greater than 40% TBSA and those with less extensive burns [80]. Such information could be useful for clinicians and thus further research efforts should be aimed at investigating the change in levels of hemostatic markers in patients with different burn sizes.

As previously discussed, traditional coagulation tests such as PT and APTT provide valuable information but often lack the sensitivity and specificity required to detect the subtle and evolving coagulation disturbances that occur in burn injuries. Further complicating the situation, coagulopathy is not static—it evolves over time. Research has demonstrated that coagulation tests taken at the scene of trauma are often not clinically significant, while measurements taken about one-hour post-injury provide more valuable insights for diagnosis and treatment [83]. This highlights the need for biomarkers that can effectively track coagulation status throughout the course of the injury. Currently, no single biomarker of coagulopathy provides a comprehensive diagnosis on its own, since each biomarker corresponds to a specific aspect of the coagulation process. With this in mind, combining multiple biomarkers may offer a more comprehensive picture of the injury and the physiological mechanisms driving it. This integrated approach could potentially lead to the identification of more sensitive biomarkers, improving our ability to diagnose and manage burn-induced coagulopathy. By continuously refining our understanding of these biomarkers and how they interact over time, clinicians can better assess and intervene in the dynamic process of coagulopathy, ultimately improving patient care.

### Biomarkers of multiple organ failure

Multiorgan failure is a severe and life-threatening complication of burn injuries, often driven by the systemic inflammation and metabolic disturbances that occur after the injury. In fact, Krishnan et al. conducted an autopsy study and found multiple organ failure to be the primary cause of over 70% of burn-related deaths [84]. Notably, the liver, heart, and kidneys are among the initial organs to fail during the progression of multiorgan failure after severe burn injuries [84]. Therefore, we will focus on these organs and the potential biomarkers that could help diagnose their failure.

### *Hepatic dysfunction*

Following severe burns, the liver becomes dysfunctional due to the direct impact of systemic inflammation, oxidative stress, and infection [7, 85]. Hepatic dysfunction following a burn can severely impair the body’s ability to combat infections and regulate the overall inflammatory response, leading to increased mortality rates [8]. Among the biomarkers used to assess hepatic injury, alanine transferase (ALT) and aspartate aminotransferase (AST) are the most sensitive indicators of hepatocyte damage [85–87]. ALT and AST, enzymes involved in amino acid metabolism, are normally present at low levels in the blood [85–87]. However, these enzymes are released into circulation following cellular injury, reflecting the extent of liver damage [85–87]. Similarly, serum alkaline phosphatase (ALKP), a serum enzyme elevated in response to thermal injury, can be used to detect hepatic dysfunction [85]. Studies have shown that serum AST, ALT, and ALKP levels increase by 50-200% post-burn when compared with normal levels after 24 and 48 h [43, 85, 86].

In an observational study of patients with TBSA burns exceeding 90%, liver dysfunction was defined as a 5-fold increase in serum ALKP from the upper limit of normal (ULN) [88]. Jeschke et al. observed that serum AST and ALT levels peaked during the first day post-burn, whereas serum ALKP peaked on the second day [86]. Interestingly, a retrospective study of 116 patients admitted to the burn unit with > 10% TBSA burned found that elevation of both AST and ALT occurred in only 41.3% of cases, with 51% of these being men [87]. The elevation of AST was seen in 26.7%, while ALT elevation was observed in just 4.3% of patients seven days following burn injury [87]. An observational study by Ketels et al. showed that serum AST and ALT were significantly more reliable in diagnosing hepatic dysfunction in burns with > 50% TBSA, with levels spiking on day one and normalizing by day three [89]. Although AST and ALT are generally considered the gold standard for measuring hepatocyte injury, they are not always reliable, as AST is also indicative of cardiac arrest and muscle injury [7].

Given that ALT and AST are less reliable in patients with lower TBSA burns, Ketels et al. conducted a pilot prospective study on 58 ICU burn patients to test the ratio of albumin to alkaline phosphatase (AAPR) in detecting burn-induced hepatic dysfunction [89]. The enrolled patients had a median age of 50 and an average TBSA burned of 9.13% [89]. While AST and ALT did not show significant changes in the first two weeks post-burn, the serum AAPR demonstrated a notable decrease over time, with a rate of -0.08/day [89]. They found that increases in serum AAPR increased the likelihood of ICU discharge, highlighting the potential of AAPR as a biomarker for burn-induced hepatic dysfunction [89].

Another alternative biomarker which addresses the lack of liver specificity in ALT and AST, is serum glutamate dehydrogenase (GLDH) [7, 90]. Schomaker et al. conducted a study of 131 subjects observing GLDH levels in patients with severe liver injury [90]. They found that serum GLDH levels greater than 2.5 times the ULN were indicative of liver injury [90]. Furthermore, the ROC curve analysis for 843 subjects demonstrated that the sensitivity and specificity of GLDH with respect to liver injury was 0.98, proving its reliability as a biomarker for hepatic dysfunction [91]. Although this study did not specifically examine GLDH in burn injury, it may still be useful as a biomarker to assess hepatocyte damage in burn patients.

Lastly, serum bilirubin levels, particularly total bilirubin (TBIL), serve as significant prognostic factors for liver dysfunction and mortality following severe burns, as peak TBIL values have been found to be significantly higher in non-survivors than in survivors [88]. Gong et al. defined liver dysfunction as a 1.5-fold increase in TBIL and found that it peaked around two weeks post-burn [88]. Lastly, serum glutathione (GSH) levels have been correlated with the severity of hepatocyte damage through a cross-sectional study of 40 burn patients investigated on the first, second, and seventh day post-admission in burn patients with > 15% TBSA, which demonstrated a significant decrease in serum GSH levels [92]. Although no significant correlation was observed between serum GSH levels and TBSA of burn injury, GSH offers insight into the extent of hepatic dysfunction and oxidative stress following severe burns [92].

### *Renal failure*

Renal failure in burn patients is a critical complication that can severely affect recovery and overall prognosis. As previously mentioned, the severe post-burn inflammatory response leads to multiple organ dysfunction, including acute kidney injury (AKI). AKI typically presents as a rapid and reversible decrease in kidney function and can be defined as an early or late stage [93]. Early AKI tends to occur in the first 24 h post-burn and can often be effectively prevented by aggressive fluid resuscitation [93]. In contrast, late AKI usually develops two to three weeks after the initial injury, which is usually due to sepsis and MODS [94]. Mosier et al. conducted a study on 221 adults with a mean TBSA burn of 42%, all of whom had no known history of chronic renal dysfunction [95]. Of these patients, 62 adults (28%) met AKI criteria using the RIFLE (Risk, Injury, Failure, Loss of kidney function, and End-stage kidney disease) classification within 24 h [95]. Among those who did not develop early AKI, 47 patients (30%) presented with AKI later in their hospitalization [95]. This study underlines the high incidence of AKI in burn patients, with nearly half developing the condition [95]. A similar study was conducted by Chung and colleagues using both Acute Kidney Injury Network (AKIN) and RIFLE criteria to evaluate AKI in 1973 patients [96]. Among those with burns covering more than 20% of their TBSA, the prevalence of AKI was 77% using the AKIN criteria, and 62% using the RIFLE criteria [96]. Palmieri et al. also studied AKI in adult burn patients, finding that AKI occurred in 32 (53.3%) of 60 patients with severe burns, according to the maximum RIFLE category [97]. While these studies highlight a substantial incidence of AKI in burn patients, Emami et al. and Stenivall et al. reported lower percentages of AKI development [98, 99]. This discrepancy may be attributed to the use of different AKI criteria (RIFLE or AKIN) across studies, as well as the varying severity of burn injuries among patient populations [99].

Early AKI biomarkers, such as serum creatinine (sCr), serum cystatin C, plasma and urine neutrophil gelatinase associated lipocalin (NGAL), have proven to be useful in predicting AKI [63]. However, sCr and cystatin C levels only rise significantly after 12 h post-admission, limiting their effectiveness as early indicators [100]. In contrast, plasma and urine NGAL levels were drastically increased at the time of admission, making this a superior biochemical marker for diagnosing early AKI, particularly in burn patients with larger TBSAs [100]. Most notably, Kidney injury molecule-1 (KIM-1), a type I transmembrane protein, was demonstrated by Ren et al. to potentially be the most stable, reliable, sensitive, and specific indicator for early diagnosis of AKI [101]. Other recent studies have also shown that the detection of KIM-1 in kidney tissue and urine facilitates the early diagnosis of AKI and is a better indicator than sCr or serum blood urea nitrogen (BUN) [101]. Burn patients who develop AKI show general biomarkers common to all AKI patients, along with specific biomarkers related to the unique pathophysiological processes involved in burns, such as systemic inflammation and hypermetabolism.

### *Cardiac dysfunction*

In 1931, Blalock suggested that impaired cardiovascular function was a major factor leading to organ failure following burn injury [102–104]. Myocardial dysfunction is generally characterized by slowed isovolumic relaxation, impaired contractility, and decreased diastolic compliance of the left ventricle [103]. This dysfunction is often manifested by decreased cardiac output, which can result from causes such as hypovolemia and cardiac stress following a burn injury [102, 103, 105, 106]. Despite recent clinical studies, the cardiovascular response to burn injuries remains poorly understood [102].

Previously, biomarkers such as lactate dehydrogenase, creatinine phosphokinase and MB isoenzyme (creatine kinase-MB; CK-MB) were used as indicators of cardiac injury [102, 107]. However, these lacked specificity for clinical use due to the significant muscle and soft tissue damage often present in burn injuries [102, 107]. Therefore, cardiac troponin-I (cTnI), a regulatory contractile protein specific to cardiac muscle, is a more superior biomarker for detection of cardiac dysfunction [102, 107, 108]. Chen et al. demonstrated that cTnI levels are detectable within the first two days post-burn and again from day five onward [106–108]. Their study, which involved 30 patients with TBSA burns ranging from 15 to 98%, each had four to six blood samples collected at two-day intervals between the 5th and 14th days post-burn [106–108]. All patients exhibited increased cTnI levels in at least two samples, with peak values occurring between 7 and 13 days post-burn, which appeared to be associated with early burn wound infection [106–108]. Additionally, cTnI levels were significantly higher in patients with TBSA burns greater than 20% [106–108]. Segura and colleagues also recorded elevated cTnI levels in adult burn patients daily [109]. They observed that cTnI levels increased directly after burn injury and levels peaked at day seven similar to Chen’s findings [109]. Overall, biomarkers for cardiac dysfunction following burn injuries are scarce, largely due to a lack of recent studies, particularly those involving human patients. This underscores a significant research gap that future studies could aim to fill.

MOF creates a physiological domino effect, where the failure of one organ triggers the failure of others. This interconnected dysfunction highlights the complexity of managing MOF in burn patients, underscoring the need for biomarkers that reflect specific organ dysfunctions, aiding in the early diagnosis of MOF. While many of the biomarkers listed above are currently used as gold standards, they clearly have limitations, particularly in their specificity and sensitivity across different populations, injury severities, and time post-injury. To improve early detection and intervention, there is a need for biomarkers that are generalizable to all burn patients, regardless of demographic or injury-related differences. Identifying biomarkers that can reliably detect the onset of organ failure before it progresses to MOF is crucial for preventing systemic collapse and improving patient outcomes.

### Biomarkers of sepsis

Infection is the most frequent complication following severe burn injury, often escalating to sepsis, then septic shock, and eventually, MODS [110]. Notably, sepsis is one of the most frequent and severe complications following burn injuries, with TBSA being the most significant risk factor for developing sepsis in burn patients [2, 111]. Indeed, sepsis is the most common cause of death amongst burn patients who survive the initial burn injury and is estimated to account for nearly two thirds of deaths among these individuals [2, 112]. Enhancing patient outcomes in acute burn care relies on early detection of infection to enable timely interventions [110]. In fact, each six-hour delay in a sepsis diagnosis decreases patient survival by 10% [113]. However, diagnosing sepsis in this demographic has proven challenging due to the burn-induced hypermetabolic response and systemic inflammation, which both can mimic and mask clinical criteria of sepsis [110]. Therefore, biomarkers could play an important role in helping reliably detect sepsis early on to administer antimicrobial therapies in a timely manner.

CRP is primarily synthesized by hepatocytes, and is stimulated by inflammatory cytokines, like IL-6, in response to tissue damage, as well as infectious stimuli [2, 113]. The literature presents conflicting evidence regarding the use of CRP as a biomarker for major infection and sepsis. However, there is evidence supporting CRP as a prognostic indicator and early predictor of sepsis in burn patients. Plasma concentrations of CRP in healthy individuals are nearly undetectable but are elevated in burn patients with infections or sepsis [2]. CRP can be detected six to eight hours after the start of infection, reaching peak concentrations 36–50 h post-burn [113]. John and colleagues found that burn-induced septic patients experienced a more rapid and earlier increase in serum CRP levels compared to non-septic burn patients, particularly when TBSA was greater than 50% [113]. In the same prospective study of 60 thermal burn patients, researchers found that an increase in serum CRP concentrations predicted sepsis with an efficacy of 87%, a sensitivity of 93%, and a specificity of 80% [113]. Further, levels of CRP could indicate sepsis approximately two days before the appearance of clinical symptoms [113]. Interestingly, similar findings were observed in a study of 57 pediatric burn patients wherein a rise in serum CRP predicted sepsis 82% of the time, with 100% sensitivity as sepsis was always preceded or accompanied by a rise in serum CRP levels [114]. Moreover, the increase in serum CRP occurred 2.3 +/- 0.5 days before clinical diagnosis [114]. However, CRP was found to have limited specificity (69%), as it can also rise due to other inflammatory events [114]. Therefore, evaluating serum CRP levels may be useful in conjunction with other clinical and laboratory markers of sepsis to enhance early detection efforts by prompting close monitoring [113, 114]. Given the conflicting data regarding the use of CRP as a predictor of sepsis in burns, a large cohort study of adult burn patients should be carried out to confirm whether this biomarker can accurately predict the incidence of sepsis in this demographic.

Recent evidence has emerged supporting the use of the serum CRP-to-albumin ratio (CAR) as a predictor of sepsis and prognostic indicator in patients with severe burn injury. Indeed, multivariate logistic regression analysis revealed that admission serum CAR and percent TBSA were independent risk factors for sepsis in these patients [115]. Notably, in this study, admission serum CAR was found to be the most significant predictor of sepsis with a cut-off value of 1.66 [115]. This threshold was associated with an AUC of 0.793, 74.34% sensitivity, and 72.29% specificity [115]. Moreover, admission serum CAR ≥ 1.66 was associated with decreased 30-day survival following a burn injury [115].

Procalcitonin (PCT) is a precursor of the hormone calcitonin and is encoded by the calcitonin-1 (CALC-1) gene [2, 116]. PCT is a well-recognized biomarker of infection and has proven effective for detecting sepsis in burn patients [116–121]. In fact, a positive correlation has been found between serum PCT levels and TBSA, a significant risk factor for developing sepsis [111, 117, 119]. Under normal conditions, PCT is produced by neuroendocrine thyroid C cells at low levels [2, 116, 120]; however, during systemic infections, expression of the CALC-1 gene is increased, resulting in elevated PCT concentration in the bloodstream [2, 116, 120]. Patient levels of PCT begin to rise just four hours after the onset of bacterial infection and peak within 12–24 h [116]. Once the infection is under control, PCT levels decrease by half every one to one and a half days [116]. Interestingly, the burn-induced inflammatory response results in a rise in PCT in the absence of infection, with levels correlating with TBSA but seldom exceeding 2 ng/mL [116]. In non-septic patients, PCT levels peak within 24–48 h, normalizing (1.0-1.5 ng/mL or less) by the third day [116]. However, in septic patients, PCT levels continue to rise and quickly attain values greater than 5 ng/mL and can even exceed 100 ng/mL [116]. Studies show that plasma and serum PCT concentrations are significantly higher in septic burn patients compared to non-septic burn patients [116–121]. Furthermore, PCT concentrations have been found to be significantly lower in patients who survive compared to nonsurvivors [111, 118, 119, 121]. Interestingly, in a study of 324 extensively burned patients, Xu et al. found that increased serum PCT concentrations in the early post-burn period could predict the onset of sepsis within 60 days of the burn [122]. Moreover, this increase in serum PCT was significantly correlated with mortality [122]. As such, PCT may serve as an early indicator of burn severity [122]. Although several studies have proposed varying cutoff PCT concentrations for sepsis diagnosis, an absolute cutoff value cannot be established due to patient-related variability in multiple factors [120]. Since patient characteristics can influence baseline serum PCT concentrations, serum PCT kinetics may serve as a more dependable marker for systemic infection than its absolute concentration [119]. Thus, PCT dynamics can still provide reliable information to clinicians, and the combination of repeated PCT measurements together with other laboratory and clinical sepsis biomarkers can strengthen sepsis diagnosis [123]. Indeed, dynamically monitoring PCT over time enhances its reliability as a predictive tool and minimizes the likelihood of false negatives and positives [120].

As discussed earlier, burn injuries trigger an inflammatory response, resulting in an increase in various cytokines, both pro- and anti-inflammatory [2]. Researchers have investigated the potential of these cytokines to enable early sepsis diagnosis following burn injury. As early as hospital admission, serum IL-6 and IL-10 levels have been found to be higher in septic burn patients than their non-septic counterparts [10]. Moreover, levels of these cytokines were found to correlate with the severity of sepsis [10]. Indeed, in Pileri and colleagues’ study of 60 adult burn patients, post-burn day three levels of IL-10 emerged as a key factor in distinguishing septic survivors from nonsurvivors [10]. Additionally, serum IL-8 levels have been found to increase following a burn injury, with levels significantly higher among septic patients [124]. Although studies on the inflammatory profile in post-burn adults are limited, several studies in the pediatric burn population have highlighted various inflammatory cytokines as potential biomarkers for sepsis in victims of burn injury. Pediatric burn patients with sepsis exhibit elevated serum levels of TNF-α and IL-6 as compared to their non-septic counterparts [2]. Moreover, in a clinical study of 468 pediatric burn patients, logistic analysis revealed an almost linear relationship between serum IL-8 levels exceeding 234 pg/ml and the occurrence of sepsis [125]. In a serum profiling study in pediatric burn patients, individuals who did not survive sepsis had significantly increased admission serum concentrations of IL-6 (15-fold), IL-8 (5-fold), IL-10 levels (15-fold), granulocyte-monocyte colony-stimulating factor (GM-CSF) (3-fold), and IFN-γ (4-fold), TNF-α (4-fold), and interleukin 12 (IL-12p70) (4-fold) compared to those who did not develop sepsis during acute hospitalization [11]. Furthermore, thermal-burn patients with a combination of elevated IL-6 and IL-12p70 concentration and decreased TNF-α at admission were found to be at a greater risk of developing and dying of sepsis [11].

As discussed, diagnosing sepsis in burn patients remains a significant challenge for clinicians. Indeed, sepsis is inherently complex, and its diagnosis in burn patients is further complicated by the burn-induced inflammatory response, which can increase the risk of false positive diagnoses [126]. Consequently, no one sepsis criterion is highly predictive of the syndrome, rather patients’ complete clinical presentation should be assessed [126]. In such cases, evaluating biomarkers of sepsis may serve to facilitate or strengthen a diagnosis. Indeed, a meta-analysis conducted by Cabral and colleagues found PCT to be a strong marker for early diagnosis of sepsis, especially when used in conjunction with clinical assessments and other sepsis biomarkers [127]. While current biomarkers of sepsis have their limitations, such as the limited specificity of CRP and cytokines, assessment of a panel of these markers may still serve to support a diagnosis. Researchers continue to explore potential biomarkers of sepsis that enable early detection, differentiate sepsis from other conditions, and predict patient outcomes. Interestingly, neutrophil function, immature granulocyte count, and levels of plasma cell-free DNA have shown promise for the early detection of sepsis in burn-injured patients [128]. Moreover, in two separate studies, Gille and colleagues demonstrated that mid-regional pro-adrenomedullin (MR-proADM) and mid-regional pro-atrial natriuretic peptide (MR-proANP) may serve as diagnostic markers for the onset of sepsis in this vulnerable demographic [129, 130]. Although these biomarkers show potential, reliable and consistent predictors of burn-induced sepsis remain elusive, underscoring the need for further research.

## Conclusions

Burn trauma results in severe complications that extend far beyond the visible damage of the skin, causing significant harm to muscles and other tissues, impacting nearly every system in the body. The challenge in treating burn injuries lies in the unique ways each patient may respond, making accurate diagnosis of the body’s internal changes difficult. In this context, biomarkers become essential, playing a critical role in early diagnosis and helping prevent further complications. Moreover, as our understanding of biomarkers deepens, they could pave the way for personalized treatment strategies, allowing clinicians to tailor interventions based on a patient’s unique physiological response.

Although numerous biomarkers have been identified and utilized in clinical practice over the years, a gap in the literature persists. Many of these biomarkers show inconsistent performance, limiting their predictive reliability. With the advent of high-throughput technologies, such as metabolomics, proteomics, and next-generation DNA and RNA sequencing, there is an opportunity to revisit these studies using more specific and sensitive methodologies. These advanced approaches could enable the identification of novel biomarkers with improved accuracy, potentially allowing for earlier prediction of these complications and more targeted interventions. Furthermore, many of the biomarkers, especially the inflammatory cytokines, although highly informative, are also inherently non-specific and participate in a wide range of biological processes. This lack of specificity emphasizes the importance of using these biomarkers in combination, rather than isolation. Leveraging advancements in artificial intelligence and machine learning could enable the development of sophisticated algorithms to detect specific patterns among these multifaceted biomarkers, potentially identifying signatures unique to these burn-induced disorders. Future studies should also consider investigating biomarkers in non-canonical tissues, such as blister fluid, as these may provide new insights into the physiological response to burns. By focusing on less traditional sources, researchers could potentially identify biomarkers that are more easily accessible and can be tested non-invasively. This approach could offer significant advantages in terms of patient comfort and ease of monitoring, ultimately contributing to more effective and timely clinical management of burn patients.

Moreover, many of these studies were constrained by small sample sizes, and a significant proportion focused predominantly on middle-aged male populations. This narrow focus limits the generalizability of the findings, raising questions about their applicability to other demographic groups. To address these limitations, it is crucial to conduct studies involving more diverse populations, including variations in age, sex, and ethnicity. Furthermore, leveraging meta-analyses could help evaluate the consistency of these biomarkers across different demographic groups, providing a more comprehensive understanding of their reliability and relevance in diverse clinical settings.

Lastly, it is unclear whether all identified biomarkers can be translated into clinical practice due to various challenges, including cost, the timing of biomarker presentation, and the efficiency of tests. Some biomarkers may appear too late to be useful, or the tests may take too long to yield results. Future research should focus on developing more efficient and accurate methods to incorporate biomarkers into clinical practice, ultimately providing greater patient care. Overall, biomarkers represent a promising direction for the future, with the potential to significantly improve outcomes for burn patients. Therefore, future research must prioritize the identification of more accurate, sensitive, and specific biomarkers for burn-related pathologies, with a particular emphasis on mortality. 

## Data Availability

No datasets were generated or analysed during the current study.

## Abbreviations

3-MTH: 3-Methylhisidine
AAPR: Albumin-to-Alkaline Phosphatase Ratio
AKI: Acute Kidney Injury
AKIN: Acute Kidney Injury Network
ALT: Alanine Transferase
ALKP: Alkaline Phosphatase
Ang-1: Angiopoietin-1
Ang-2: Angiopoietin-2
APP: Acute Phase Protein
APR: Acute Phase Response
APTT: Activated Partial Thromboplastin Time
AST: Aspartate Aminotransferase
BUN: Blood Urea Nitrogen
C1-INH: C1 Inhibitor
CALC-1: Calcitonin-1
CK-MB: Creatine Kinase-MB
CRP: C-Reactive Protein
cTnI: Cardiac Troponin-I
DAMP: Damage-Associated Molecular Pattern
DD: D-Dimer
F1.2: Prothrombin Fragment
FFA: Free Fatty Acid
GLDH: Glutamate Dehydrogenase
GM-CSF: Granulocyte-Monocyte Colony-Stimulating Factor
GSH: Glutathione
HMGB1: High Mobility Group Box 1
ICU: Intensive Care Unit
IL-1β: Interleukin-1β
IL-6: Interleukin-6
IL-8: Interleukin-8
IL-10: Interleukin-10
IL-12p70: Interleukin-12
IL-18: Interleukin-18
IFN-γ: Interferon γ
KIM-1: Kidney Injury Molecule-1
LBM: Lean Body Mass
MCP-1: Monocyte Chemoattractant Protein-1
MODS: Multiorgan Dysfunction Syndrome
MOF: Multiple Organ Failure
MR-pro ADM: Mid-Regional Pro-Adrenomedullin
MR-proANP: Mid-Regional Pro-Atrial Natriuretic Peptide
NGAL: Neutrophil Elatinase Associated Lipocalin
NLR: Neutrophil-Lymphocyte Ratio
PAI-1: Plasminogen Activator Inhibitor 1
PAMP: Pathogen-Associated Molecular Pattern
PC: Protein C
PCT: Procalcitonin
PS: Protein S
PT: Prothrombin Time
REE: Resting Expenditure Rate
RIFLE: Risk, Injury, Failure, Loss of kidney function, and End-stage kidney disease
sCr: Serum Creatinine
SIR: Systemic Inflammatory Response
SIRS: Systemic Inflammatory Response Syndrome
sTM: Soluble Thrombomodulin
TAT: Thrombin-Antithrombin
TBIL: Total Bilirubin
TBSA: Total Body Surface Area
TFPI: Tissue Factor Pathway Inhibitor
TG: Triglyceride
TNF-⍺: Tumor Necrosis Factor-⍺
t-PA: Tissue Plasminogen Activator
ULN: Upper Limit of Normal
VIIc: Factor VII
VIIa: Activated Factor VII
VIIc: Procoagulant Factor VII
VEGF: Vascular Endothelial Growth Factor
WAT: White Adipose Tissue
WBC: White Blood Cell
